# Supplementary figures and images for: RAR Inhibitors Display Photo-Protective and Anti-Inflammatory Effects in A2E Stimulated RPE Cells In Vitro through Non-Specific Modulation of PPAR or RXR Transactivation
Source: Int J Mol Sci. 2024 Mar 6;25(5):3037. doi: 10.3390/ijms25053037 (PMC10932305; doi:10.3390/ijms25053037)

Supplementary figure 1: Norbixine : BCL2 and GAPDH blots.

A

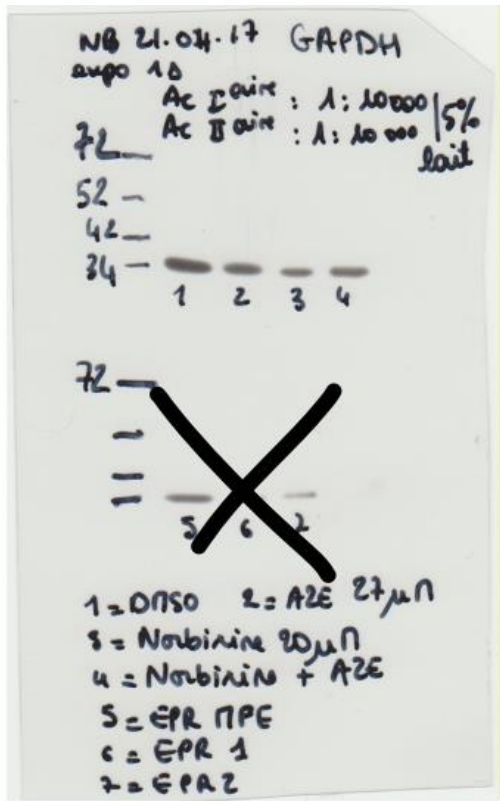

B

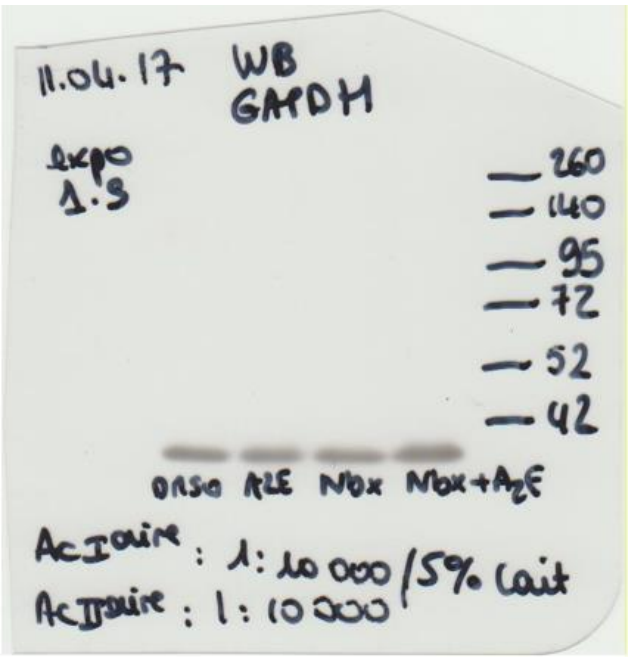

C

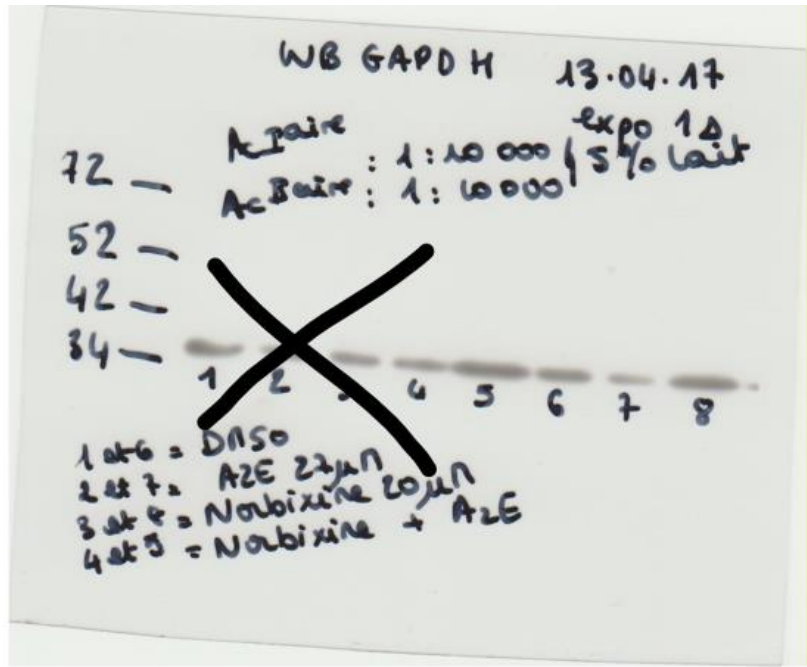

D

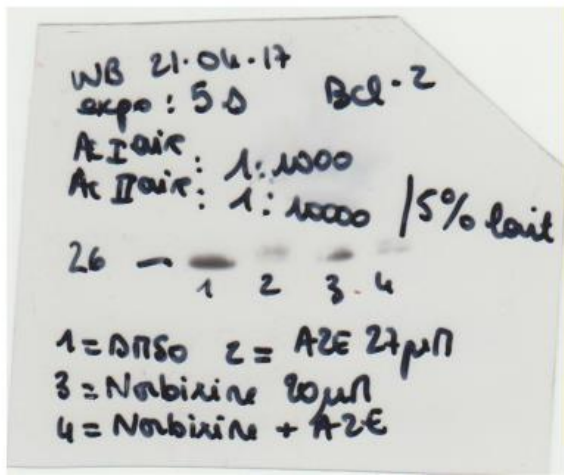

E

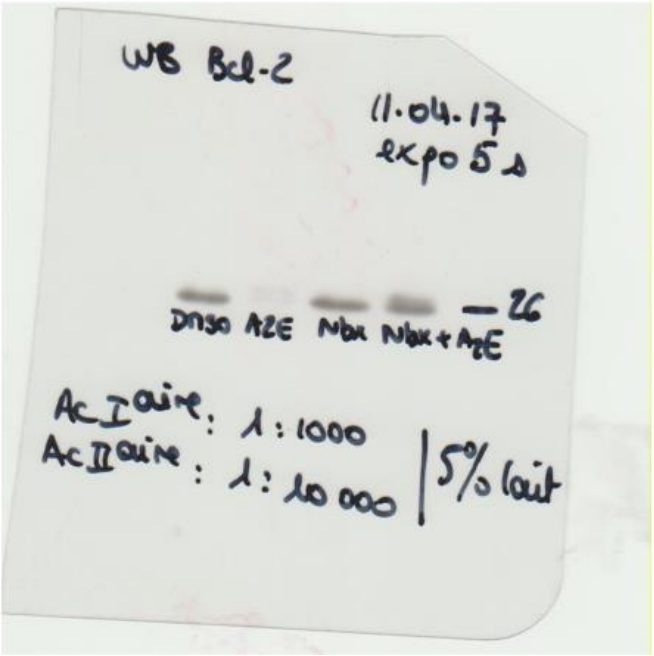

F

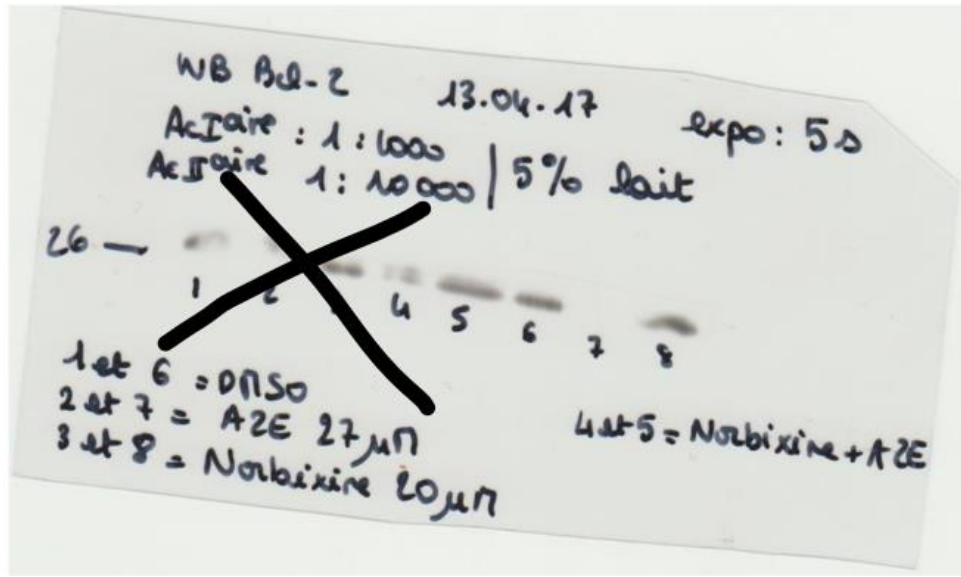

Supplement: Supplementary file 1 [file ijms-25-03037-s001.zip › ijms-2809518-supplementary/Suplementary Figure S1 BCL2 with A2E and Norbixine NBX.pdf]

Figure S2 B19 BCL2 and GAPDH blots

GAPDH blots

A

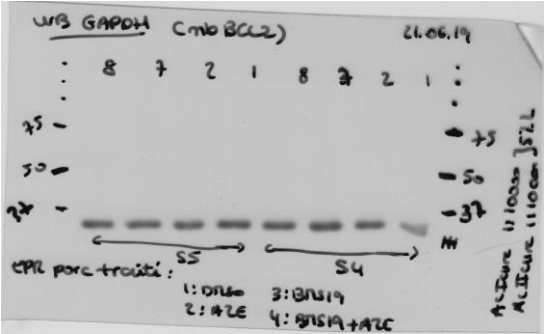

B

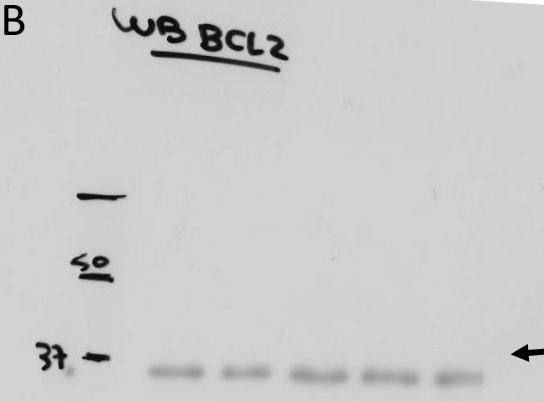

GAPDH

C

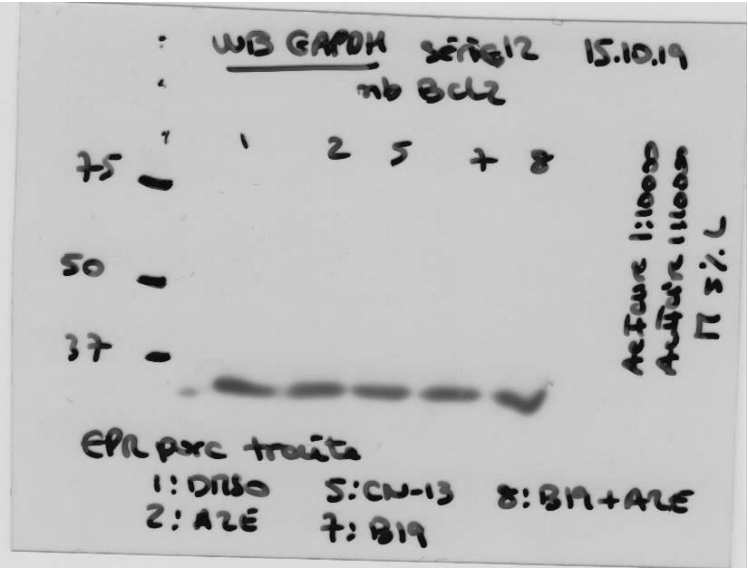

Bcl2 blots

D

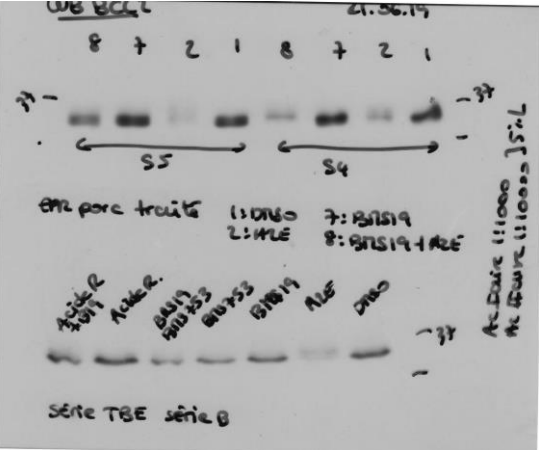

E

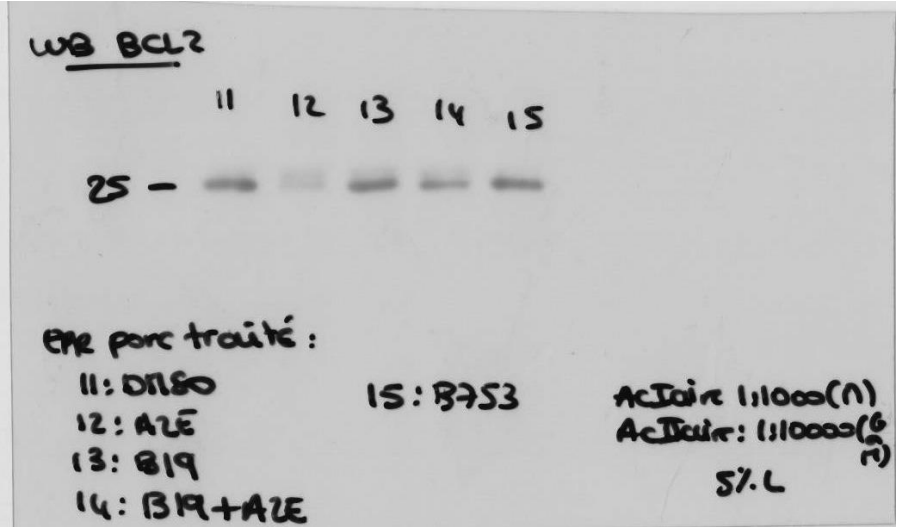

F

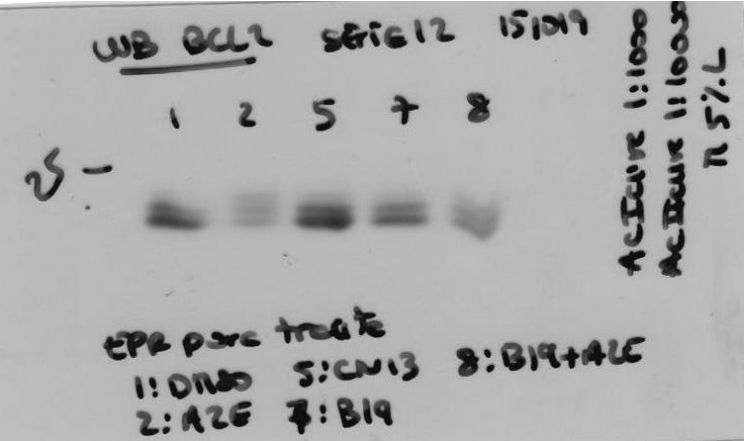

Supplement: Supplementary file 1 [file ijms-25-03037-s001.zip › ijms-2809518-supplementary/Suplementary Figure S2 BCL2 with A2E and B19.pdf]
